# Anhysteretic Magnetization Measurement Methods for Soft Magnetic Materials

**DOI:** 10.3390/ma11102021

**Published:** 2018-10-18

**Authors:** Michał Nowicki

**Affiliations:** Institute of Metrology and Biomedical Engineering, Warsaw University of Technology, 02-525 Warsaw, Poland; nowicki@mchtr.pw.edu.pl; Tel.: +48-690-650-386

**Keywords:** soft magnetic materials, Anhysteretic Magnetization, hysteresisgraph

## Abstract

This article is concerned with the methods for experimentally determining the Anhysteretic Magnetization curve for soft magnetic materials. A new method based on the modern hysteresisgraph system is presented. Known modern and traditional methods based on fluxmeters are presented as well. The experimental results obtained with the described methods for isotropic Mn–Zn ferrite are compared. Lastly, results of validation on NANOPERM^®^ nanocrystalline material are detailed and show negligible hysteresis. The new method allows for accurate Anhysteretic Magnetization curve measurement without software or hardware modifications of standard, commercially available hysteresisgraph systems. The speed and accuracy of the results are improved in comparison with other methods.

## 1. Introduction

**Figure 1:**
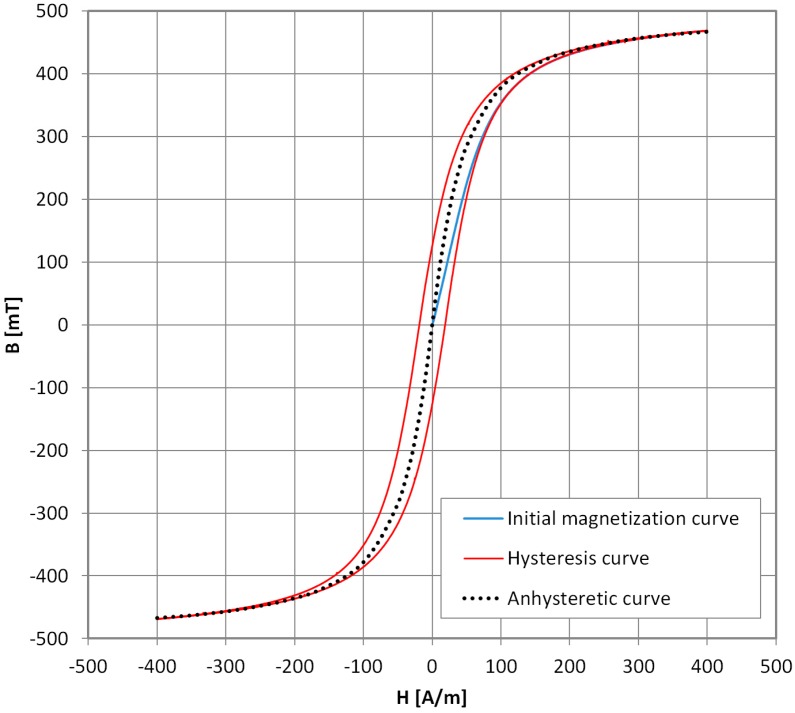
Exemplary results of measurement of initial magnetization curve (**blue line**), hysteresis curve (**red line**), and Anhysteretic Magnetization (AM) curve (**black dots**) for modern isotropic soft magnetic material (Mn–Zn-based ferrite F3001).

The Anhysteretic Magnetization curve (AM), also called ‘ideal magnetization’ when it was first introduced [1,2], is widely used in material characterization and technical applications. In Figure 1, the exemplary initial and the Anhysteretic Magnetization curve of Mn–Zn ferrite for power applications are presented. Historically, AM was used extensively in magnetic recording to overcome hysteresis of the recording medium [3], and in ‘forced’ magnetization of hard magnetic materials [4,5]. Nowadays, the AM curve concept is most often encountered in the Jiles–Atherton model of magnetic hysteresis [6]. It is one of the most popular hysteresis models, suitable for design and simulation of electronic and electrotechnical components with soft or semi-hard magnetic cores [7]. However, actual measurements of the Anhysteretic Magnetization curve are very rarely done with modern equipment, due to both the technical difficulties [8] and long measurement time. Moreover, in the literature, there are discrepancies between the definitions of the AM curve. It is described as the magnetization that would occur if the material had no imperfections [9]. In another approach, it represents the thermal equilibrium state of the material. The second definition seems more justified for use in the J–A model, which predicts that the stress-induced changes in magnetization tend towards the AM curve [10]. This is because the cyclic application of stress releases domain walls from pinning sites, and after each stress cycle, the system should adopt a state nearer thermal equilibrium [11]. Thus, AM is defined as the ‘thermal equilibrium’ curve or ‘locally demagnetized’ curve and measured by cooling the sample from above the Curie temperature in an incrementally increased direct current DC field, or during DC biased alternating current AC field demagnetization. Consequently, the results under the two definitions differ [11]. Unfortunately, the physically justified thermal method cannot be used for modern magnetic materials, such as amorphous and nanocrystalline alloys. Any phase-change or stress-relaxation effects must be avoided in order to maintain the magnetic parameters of the given sample.

Presently, the AM curve is often obtained indirectly by mathematical optimization of the J–A (Jiles–Atherton) model parameters on the basis of experimentally obtained hysteresis loops [8]. The optimization is a challenging and time-consuming numerical task [12]. There is also an open question about the validity of obtained results, as different J–A model variations give slightly different results [13].

Therefore, in this paper, a new method of AM measurement is presented, based on the standard, unmodified modern hysteresisgraph system. The method does not rely on software or hardware modifications, allowing AM measurements using commercially available systems. The traditional [5] and modern [14] methods based on analog and digital fluxmeters are also described, and the results obtained for Mn–Zn ferrite are compared. Lastly, the results of validation on novel nanocrystalline material (NANOPERM^®^) are detailed and show negligible hysteresis.

## 2. Materials and Methods

### 2.1. Traditional AM Measurement Method

**Figure 2:**
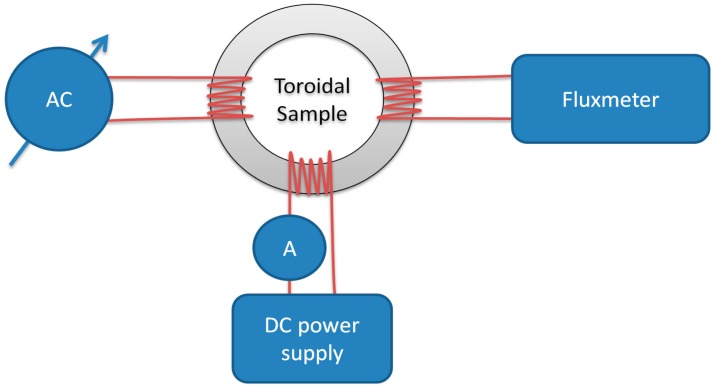
Schematic diagram of traditional AM measurement system.

Traditionally, the AM curve is obtained point-by-point by an AC field ‘demagnetizing’ the sample under an additional, incrementally increasing DC field. In Figure 2, a schematic diagram of this measurement system is shown.

The measured sample should preferably be ring-shaped/toroidal or in Epstein frame to obtain a near-homogenous magnetizing field distribution and closed magnetic path. This helps reduce demagnetizing field-induced distortion of the measurement results. Typically, for AM measurement, three coils are wound on the sample.

The first coil is connected to a variable AC current source, which can be either a Variac or signal generator with high current output. It is used to effect a steadily decreasing AC magnetizing field on the sample to ‘demagnetize’ it. The second coil is connected to a DC current source (laboratory power supply) and ammeter (A) for setting the biasing DC magnetizing field. The third measurement coil is connected to a fluxmeter of a Grassot, electromagnetic, or preferably modern electronic design. The fluxmeter used in the presented investigation was a Lakeshore 480 calibrated according to the manufacturer [15] and checked with steel ring standard samples [16].

**Figure 3:**
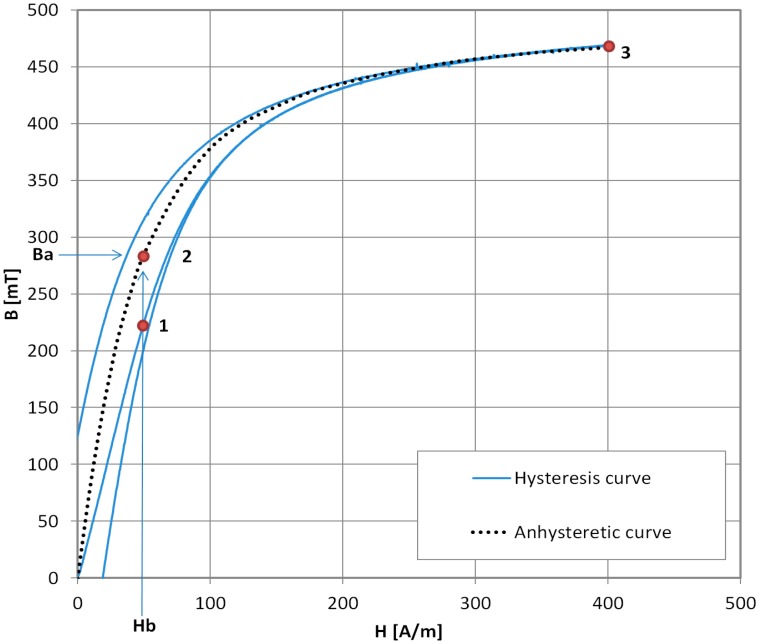
Schematic sequence of traditional point-by-point Anhysteretic Magnetization curve measurement. 1—setting *H_b_* (DC biasing field), 2—local demagnetization, 3—setting DC saturation field.

In Figure 3, the three steps of AM measurement are presented. The prepared sample is magnetized with the DC biasing magnetizing field *H_b_*. It is calculated from the ammeter current *I* reading with the formula:

(1) $$H_{b}=\frac{nI}{L}$$

where *H_b_* is the magnetizing field, *n* the number of windings, *I* = current, and *L* = magnetic path. Thus, point ‘1’ is reached on the initial magnetization curve of the given material. Next, the variable AC current source is used to locally ‘demagnetize’ the sample by applying the AC magnetizing field. Its initial amplitude should be at least 2 times greater than *H_s_*—the saturation magnetizing field. After local ‘demagnetization’, the sample is in the ‘2’ state—the point with coordinates (*B_a_*, *H_b_*), where *B_a_* is the value of anhysteretic magnetization for a given *H_b_* biasing magnetizing field. To correctly infer the *B_a_* value, the fluxmeter is then zeroed and adjusted for drift, if possible. The DC magnetizing field is then set to the *H_s_* value in order to saturate the sample. The sample reaches saturation point ‘3’ with saturation induction *B_s_*, and the fluxmeter reading is *B*_1_. The *B_a_* value is found simply:

(2) $$B_{a}=B_{s}-B_{1}$$

To obtain the full AM curve *B_a_* = *f*(*H_b_*), steps 1–3 are repeated for the incrementally increasing DC biasing field *H_b_*.

The saturation magnetizing field *H_s_* and saturation induction *B_s_* of the sample are found in the usual way with a fluxmeter and suitable magnetizing field source, e.g., by the method of reversals.

### 2.2. Modern AM Measurement Method

**Figure 4:**
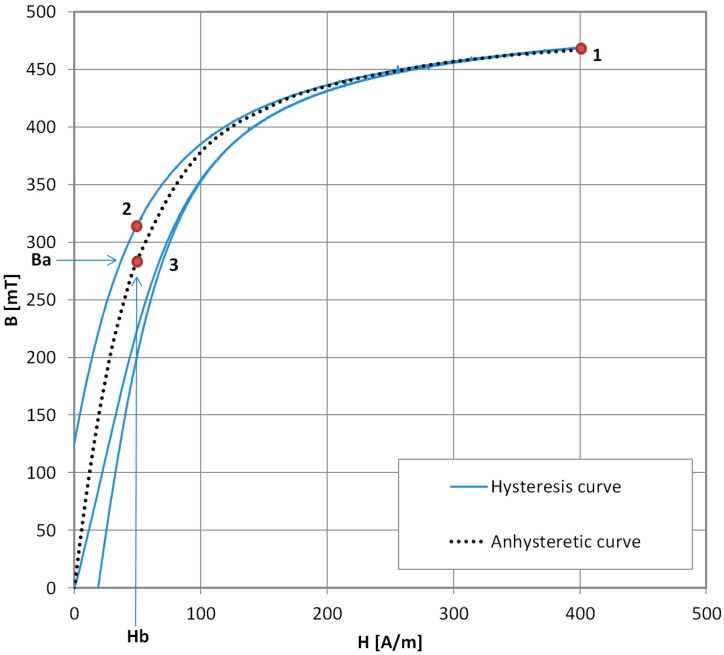
Schematic sequence of modern point-by-point Anhysteretic Magnetization curve measurement. 1—setting the DC saturation field, 2—setting *H_b_* (DC biasing field), 3—local demagnetization.

In modern AM measurements, which can be found in recent literature [17], the measurement is performed automatically under proper software control, but the schematic diagram of the measurement stand is the same as in Figure 2. The measurement sequence is changed, and it is presented in Figure 4.

The sequence is as follows. First, the DC saturation magnetizing field *H_s_* is set, and the saturation induction *B_s_* in the is sample reached (point ‘1’). The fluxmeter is then zeroed, and the *H* field value is changed to biasing field value *H_b_* (reaching point ‘2’ on the characteristic). This is immediately followed by local ‘demagnetization’ by the AC magnetizing field. The sample reaches point ‘3’, which is on the AM curve. The fluxmeter reading is *B*_2_. To obtain the AM value:

(3) $$B_{a}=B_{s}-B_{2}$$

To obtain the full AM curve *B_a_* = *f*(*H_b_*), steps 1–3 are repeated for the incrementally increasing DC biasing field *H_b_*.

This method has the obvious disadvantage of longer fluxmeter measurement time (most, if not all, DC fluxmeters are prone to time drift errors) and the need to integrate induction *B* values during the local AC field ‘demagnetization’. However, the (*H_s_*, *B_s_*) reference point in this method is definite, and the fully automated systems presented in literature have good repeatability.

### 2.3. Novel Hysteresisgraph AM Measurement Method

**Figure 5:**
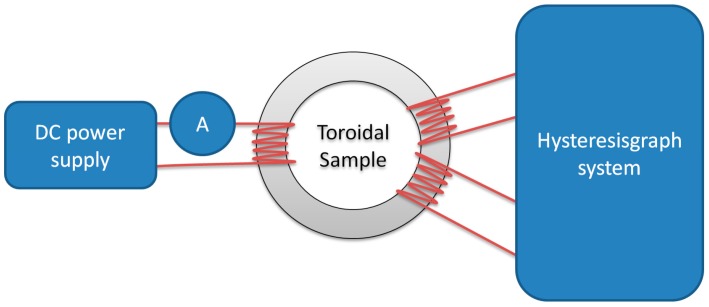
Schematic diagram of a novel AM measurement method.

In order to raise the accuracy of the AM results while lowering the time and manual labor needed for a single AM curve determination and permitting the use of modern magnetic measurement equipment, a new method is described. The schematic diagram of the measurement stand is presented in Figure 5. The measurement stand consists of the modern hysteresisgraph system [18], equivalent to commercially available ones. The necessary condition for the hysteresisgraph system is the possible measurement of the initial magnetization curve. There is no software modification needed, and the only additional hardware is the DC power supply (DF173003C, NDN, Warsaw, Poland) and ammeter (TH1961, Tonghui, Changzhou, China). As before, the ring-shaped sample should have three windings—standard magnetizing and measurement windings connected to the hysteresisgraph system, and additional winding connected to the DC power supply for biasing the DC magnetizing field *H_b_* setting.

**Figure 6:**
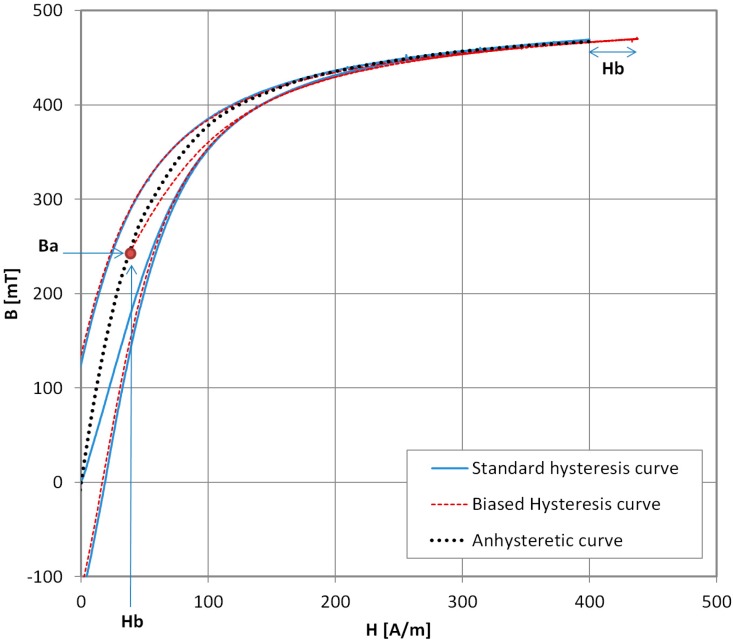
Schematic of the new AM measurement methodology. The Anhysteretic Magnetization *B_a_* value for a given magnetizing field *H_b_* is the starting point of the initial magnetization curve under the influence of a biasing field.

In Figure 6, the measurement sequence is presented. First, the biasing DC magnetizing field *H_b_* is set with the power supply. Next, the hysteresisgraph system demagnetizes the sample and plots the hysteresis curve together with the initial magnetization curve. The amplitude of the magnetizing field should be enough to fully saturate the sample, i.e., *H_s_*.

The point (*B_a_*, *H_b_*) on the Anhysteretic Magnetization curve is the first point of the initial magnetization curve measured under a DC bias. The *H_b_* value is the biasing field set with the DC power supply and is calculated with Equation (1). Due to the system drift-adjusting and loop-centering algorithms, the value of *B_a_* is also not to be read directly from the plots as the starting initial magnetization curve value *B*_0_. It is obtained as follows. Two additional hysteresis loop measurements are needed—first is the loop measurement for the *H_s_* magnetizing field, without the biasing field, to obtain the peak, saturated value of *B_s_*. It is enough to measure once for the given sample. Secondly, after each *H_b_* biased measurement is performed with the *H_s_* magnetizing field amplitude, an *unbiased* loop is measured with *H_s+b_* = *H_s_* + *H_b_* magnetizing field amplitude to obtain the peak *B_s+b_* value. Theoretically, *B_s+b_* = *B_s_* + *µ*_0_*H_b_*, which can be calculated easily, but the accuracy is better for the measured values. The value of *B_a_* is then:

(4) $$B_{a}=B_{s+b}-\left(B_{s}-B_{0}\right)$$

Again, to obtain the full AM curve *B_a_* = *f*(*H_b_*), the sequence of the *B*_0_ and *B_s+b_* measurements is repeated for the incrementally increasing DC biasing field *H_b_*.

It should be highlighted that, despite the apparent increase in complexity, the amount of manual labor for the unautomated AM measurements is reduced, as the amount of individual manual magnetizing/biasing *H* field settings is reduced by half (no *H_s_* setting needed). Modern hysteresisgraph systems allow also for better accuracy and repeatability of the peak *B* field measurements than fluxmeters alone by measuring and averaging multiple hysteresis loops, applying drift-cancellation procedures, and filtering the results.

### 2.4. Investigated Samples

The presented measurement methods were used for the investigation of AM curves of an exemplary modern isotropic soft magnetic material. The sample used was Mn–Zn ferrite F3001 for power applications, produced by Polfer. It has a toroidal shape, with uniformly wound magnetizing, sensing, and biasing windings of *n* = 20 turns each. The AM curves were measured for a biasing field range of *H_b_* = 0–400 A/m, while the saturation *H_s_* field was set at 1000 A/m.

It is important for all of the described methods that the *H_s_* field is significantly bigger than the maximal *H_b_* biasing field.

Additionally, the new method was validated on nanocrystalline NANOPERM^®^ material, produced by Magnetec GmbH [18]. It is a highly anisotropic material with negligible hysteresis. Due to a lack of measurable hysteresis, the same cores were used previously for the validation of the J–A model of the anhysteretic magnetization curve [8]. Without distinct hysteresis, the hysteresis curve measured in the usual way must overlap with the AM curve. The AM curves were measured for a biasing field range of *H_b_* = 0–1500 A/m, while the saturation *H_s_* field was set at 3000 A/m.

## 3. Results

### 3.1. AM Measurement Results for Different Methods

**Figure 7:**
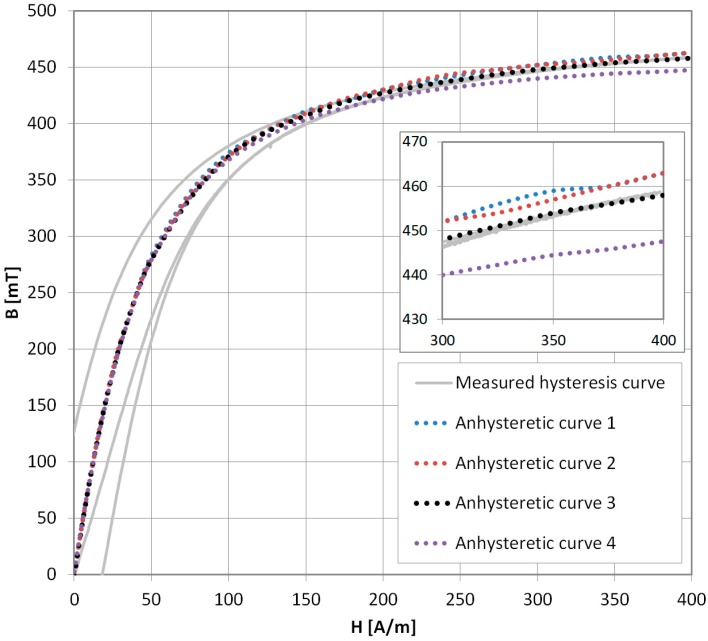
Measurement results of Anhysteretic Magnetization for F3001 ferrite. Anhysteretic curve 1—traditional method, 2—modern method, 3—new method (black dots), 4—new method without adjustment. Inset—differences in the saturation region. As can be seen, the new method is in good agreement with the measured hysteresis curve in the saturation region.

The presented methods of AM curve measurement were used on the ferrite F3001 sample. In Figure 7, results for various methods are compared by superimposition on a hysteresis loop with the initial magnetization curve measured on the hysteresisgraph system [19]. Anhysteretic curve 1 was obtained with the traditional method. Anhysteretic curve 2 was measured with the modern method, and, surprisingly, the two curves almost completely overlap.

Anhysteretic curve 3 (black dots) is the result of the new method presented in this paper. Results almost overlap with those obtained with the other methods, but in the saturation region (Figure 7, inset) they fit the hysteresis loop better. The older methods show about 1% overshoot. Additionally, anhysteretic curve 4 is constructed directly from the *B*_0_ values, omitting the adjustment described by Equation (4). While the results should be correct, the error is significant (maximum 3% in saturation). Thus, the unadjusted method can be used only for faster, qualitative measurements.

### 3.2. Validation of the New Method

**Figure 8:**
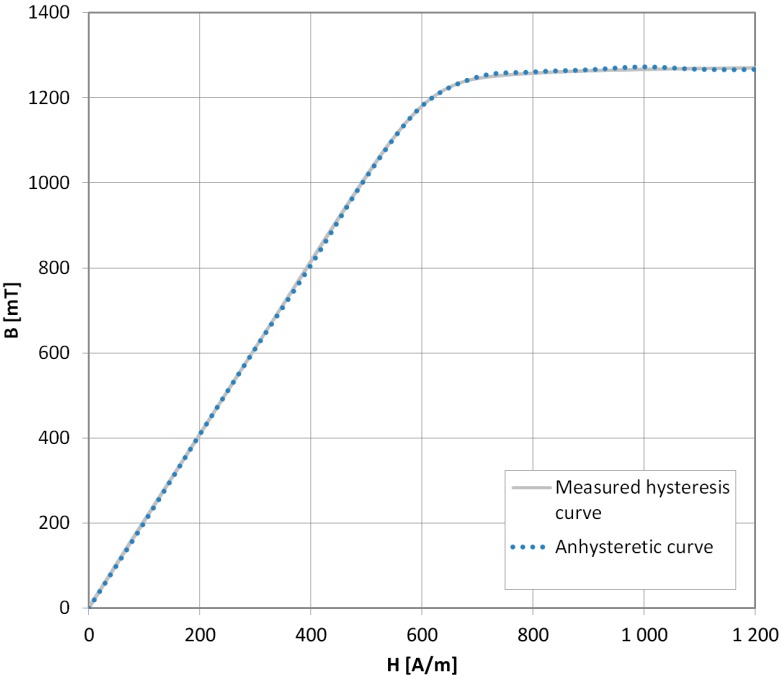
Anhysteretic magnetization curve measurement results (blue dots) and hysteresis loop (gray line) for NANOPERM^®^ nanocrystalline alloy. Very good agreement of the results is shown.

In order to validate the new method further, the AM curve was measured for the NANOPERM^®^ nanocrystalline material, which exhibits almost no hysteresis behavior. Therefore, according to [8], the anhysteretic curve and hysteresis loop should overlap. The results presented in Figure 8 confirm this.

## 4. Conclusions

The presented results of exemplary isotropic material AM curves measurements confirm the correctness of the new AM measurement method. The results are directly comparable with those obtained using traditional and modern fluxmeter-based methods. Additionally, the errors in the saturation region are lower, and due to better measurement of the *B* field peak values by hysteresisgraph systems, the scatter of the results and overall repeatability is improved.

What is not so obvious from the results is the reduced time and labor needed for the manual AM curve measurement. The presented fluxmeter-based measurements took about 1 h each, while with the new method, it was about 30 min (time varies significantly depending on measurement and demagnetization parameters). Of course, such time and labor costs are hardly acceptable for regular work, and future works are oriented toward full automation of the measurement system. This will allow for broad investigation of stress and temperature influence on the Anhysteretic Magnetization curves of soft magnetic materials, especially in context of the Jiles–Atherton model.

The main objective of the paper, however, is to present traditional, modern, and new anhysteretic magnetization curve measurement methods in a way that allows for AM investigations with modern equipment, especially self-contained, commercial hysteresisgraph systems, without the need for software or hardware modifications.
